# Outcomes after delivery room positive pressure ventilation in late preterm and term infants

**DOI:** 10.1016/j.resplu.2024.100670

**Published:** 2024-05-31

**Authors:** Maureen Peers de Nieuwburgh, Charlotte Cecarelli, Danielle Weinberg, Kesi C. Yang, Heidi M. Herrick, Elizabeth E. Foglia

**Affiliations:** a Department of Pediatrics, Cliniques Universitaires Saint-Luc, Avenue Hippocrate 10, 1200 Brussels, Belgium; b Division of Neonatology, Perelman School of Medicine, University of Pennsylvania 8 Ravdin, 3400 Spruce Street, Philadelphia, PA 19104, USA

**Keywords:** Resuscitation, Positive-pressure ventilation, Infant, Birth, Neonatology, Post-resuscitative care

## Abstract

**Objectives:**

Characterize short-term outcomes of late preterm and term infants who received positive pressure ventilation in the delivery room and compare these with infants who did not receive resuscitation at birth.

**Study Design:**

Single center retrospective cohort study of infants born between 35 0/7 and 41 6/7 weeks’ gestation in 2019. Baseline characteristics and outcomes of infants who received positive pressure ventilation were compared with controls who did not receive delivery room ventilation. The primary outcome was neonatal intensive care unit admission; secondary outcomes included multiple hospital morbidities and interventions.

**Results:**

Among 202 infants who received delivery room positive pressure ventilation, 77 (38.1%) received ≤1 min, and 125 (61.9%) received >1 min of positive pressure ventilation. Neonatal intensive care unit admission directly following resuscitation was more common in the ventilation cohort (33%) compared with controls (1.5%), *p* ≤ 0.0001. After initial admission to the newborn nursery, intensive care unit transfer rates were similar in the positive pressure ventilation cohort (4%) and controls (5%). Antibiotic exposure, hypoxic ischemic encephalopathy, respiratory support in the neonatal intensive care unit, and pneumothorax were more common in the ventilation cohort. The composite outcome of any post-delivery complication occurred in 45% of positive pressure ventilation-exposed infants, compared to 15.8% of control infants (<0.0001); this was more common following >1 min (52.8%) than ≤1 min positive pressure ventilation (32.5%), *p* = 0.002.

**Conclusion:**

Post-delivery complications are common following delivery room positive pressure ventilation, emphasizing the need for post-resuscitation monitoring in either the neonatal intensive care unitor newborn nursery setting.

## Highlights

• We assessed post-resuscitation outcomes for late preterm and term newborns.
• Postnatal complications rates are higher following longer duration of resuscitation.
• NICU admission is common immediately following delivery room resuscitation.
• Among newborns admitted to the nursery after resuscitation, ICU transfer is rare.
• These findings may inform post-resuscitation monitoring protocols.

## Introduction

At birth, newborn infants transition from liquid-filled to air-filled lungs. This process of lung aeration is critical to provide a source of gas exchange after delivery and triggers the successful adaptation to extra-uterine life^1^, ^2^. While most newborn infants achieve lung aeration through spontaneous breathing, 3–10% of all newborn infants^3^, ^4^, ^5^, ^6^ receive respiratory interventions to support this process, and 1%^7^, ^8^ receive intense resuscitation (such as intubation and invasive ventilation) at birth. For infants who do not breathe spontaneously, neonatal resuscitation guidelines recommend initiating positive pressure ventilation (PPV) within the first 60 seconds after birth^9^.

Between 3% and 10% of term and late-preterm babies receive delivery room (DR) PPV^4^, ^5^, ^10^. Infants who receive DR resuscitation at birth may experience ongoing physiologic instability after resuscitation. Potential challenges include glucose instability, electrolyte abnormalities, and respiratory compromise. Because of these possible complications, American Academy of Pediatrics/American Heart Association (AAP/AHA) neonatal resuscitation guidelines recommend post-resuscitative monitoring for infants who receive DR resuscitation^9^. However, little evidence is available to guide clinical decision-making around the most appropriate location and monitoring after DR resuscitation, particularly for infants who receive transient DR PPV and subsequently demonstrate normal vital signs. To fill this gap, a better understanding of the natural history of post-resuscitative outcomes following PPV is needed.

The objective of the current study was to characterize short-term outcomes of late preterm and term infants who received PPV in the DR and compare them to infants who did not require any type of resuscitation at birth. In addition, short-term outcomes of late preterm and term infants who receive PPV in the DR were also characterized depending on PPV duration in the DR.

## Methods

This retrospective cohort study was reviewed by the Institutional Review Board (IRB) of the University of Pennsylvania and deemed exempt from IRB oversight.

### Population

The study included all newborn infants born between 35 0/7 and 41 6/7 weeks’ gestation at the Hospital of the University of Pennsylvania (HUP) between January 1, 2019, and December 31, 2019 who received PPV in the DR. We excluded infants with a birthweight below 2 kg (as these patients are all admitted to the NICU based on hospital policy), maternal opioid use during pregnancy, triplets or higher order multiples, major congenital anomalies and absence of PPV duration in the medical chart. For each infant in the PPV cohort, we identified a single control who did not receive any DR respiratory support from the same gestational age range (35 0/7–41 6/7 weeks’ gestation) as the study cohort with the same biological sex, mode of delivery, and date of birth (within two calendar days, if no control was born on the same day – Table 1).

**Table 1:** Baseline Characteristics.

Maternal characteristics	PPV (*n* = 202)	Controls (*n* = 202)	*P*-value	PPV ≤ 1 min (*n* = 77)	PPV > 1 min (*n* = 125)	*P*-value
Maternal age, years (mean, SD)^1^	28.9 (6.5)	29.9 (5.9)	0.1	29.4 (7.1)	28.6 (6.0)	0.40
Maternal history						
Hypertension, *n* (%)	55 (27.1%)	47 (23.2%)	0.36	4 (5.2%)	11 (8.8%)	0.34
Diabetes (all types), *n* (%)	25 (12.4%)	27 (13.4%)	0.77	4 (5.2%)	2 (1.6%)	0.14
Obesity, *n* (%)	30 (14.8%)	33 (16.3%)	0.68	10 (13%)	20 (16%)	0.56
Thyroid dysfunction, *n* (%)	8 (4%)	18 (8.9%)	0.04	4 (5.2%)	3.20% (4)	0.48
Infant characteristics						
Male sex, *n* (%)^2^	108 (53.5%)	108 (53.5%)	>0.99	41 (53.3%)	67 (53.6%)	0.96
Gestational age, weeks (median, IQR)^2^	39.1 (37.5, 40.3)	39.0 (38, 39.7)	0.52	39.1 (37.9, 40.3)	39 (37.3, 40.3)	0.39
Birth weight, grams (mean, SD)^3^	3233 (573.2)	3211 (488.8)	0.69	3248 (574.2)	3223 (574.6)	0.77
Race			0.35			0.07
Black/African-American, *n* (%)	136 (67.3%)	134 (66.3%)		47 (61%)	89 (71.2%)	
White, *n* (%)	33 (16.3%)	46 (22.8%)		10 (13%)	23 (18.4%)	
Asian, *n* (%)	13 (6.4%)	9 (4.5%)		8 (10.4%)	5 (4%)	
Unknown, *n* (%)	10 (5%)	7 (3.5%)		6 (7.8%)	4 (3.2%)	
Other, *n* (%)	10 (5%)	6 (3%)		6 (7.8%)	4 (3.2%)	
Ethnicity			0.69			0.93
Hispanic, *n* (%)	5 (2.5%)	8 (4%)		2 (2.6%)	3 (2.4%)	
Twin, *n* (%)	10 (5%)	5 (2.5%)	0.19	5 (6.5%)	5 (4%)	0.43
Pregnancy and Delivery characteristics						
Pregnancy complication, *n* (%)	24 (11.9%)	37 (18.3%)	0.07	10 (13%)	14 (11.2%)	0.70
Maternal medication associated with neonatal hypoglycemia, *n* (%)	38 (18.8%)	35 (17.3%)	0.70	15 (19.5%)	23 (18.4%)	0.85
Maternal positive GBS, *n* (%)	56 (27.7%)	55 (27.2%)	0.91	23 (29.9%)	33 (26.4%)	0.59
Chorioamnionitis, *n* (%)	36 (17.8%)	11 (5.5%)	<0.001	12 (15.6%)	24 (19.2%)	0.51
ROM > 24 h, *n* (%)	16 (7.9%)	13 (6.4%)	0.56	5 (6.5%)	11 (8.8%)	0.56
Meconium-stained amniotic fluid, *n* (%)	48 (23.8%)	14 (6.9%)	<0.001	22 (28.6%)	26 (20.8%)	0.21
General anesthesia, *n* (%)	12 (5.9%)	4 (2%)	0.04	2 (2.6%)	10 (8%)	0.12
Presence of neonatal providers at birth, *n* (%)	201 (99.5%)	138 (68.3%)	<0.001	76 (98.7%)	125 (100%)	0.20
Vaginal delivery, *n* (%)^2^	90 (44.5%)	90 (44.5%)	>0.99	34 (44.2%)	56 (44.8%)	0.93
Induced vaginal delivery, *n* (%)	34 (37.8%)	12 (13.3%)	<0.001	11 (32.35%)	23 (41.07%)	0.41
Instrumentation, *n* (%)	21 (10.4%)	3 (1.5%)	<0.001	8 (10.4%)	13 (10.4%)	>0.99
C-Section, *n* (%)	112 (55.5%)	112 (55.5%)	>0.99	43 (55.8%)	69 (55.2%)	0.93
Elective, *n* (%)	30 (14.9%)	55 (27.2%)	0.002	16 (20.8%)	14 (11.2%)	0.06
Urgent, *n* (%)	82 (40.6%)	57 (28.2%)	0.009	27 (35.1%)	42 (33.6%)	0.83
Nuchal cord, *n* (%)	46 (22.8%)	21 (10.4%)	<0.001	19 (24.7%)	27 (21.6%)	0.61
Umbilical cord gas pH, (median, IQR)^4^	7.2 (7.1, 7.2)	7.3 (7.2, 7.3)	<0.001	7.2 (7.1, 7.3)	7.2 (7.1, 7.2)	0.37
Apgar score at 1 MOL, (median, IQR)^5^	3 (2, 5)	8 (8, 9)	<0.001	5 (3, 6)	2 (2, 4)	<0.001
Apgar score at 5 MOL, (median, IQR)^5^	8 (6, 9)	9 (9, 9)	<0.001	9 (8,9)	7 (5, 8)	<0.001
Sepsis Risk Score, (median, IQR)	0.2 (0.06, 0.4)	0.09 (0.05, 0.2)	<0.001	0.2 (0.05, 0.4)	0.2 (0.07, 0.5)	0.36

*Abbreviations:* PPV: Positive pressure ventilation, SD: Standard deviation, IQR: Interquartile range, GBS: group B streptococcus, ROM: Rupture of the membranes IQR: Interquartile range, MOL: minutes of life.

1 Out of 202 infants in the PPV group, maternal age value was available for 200 infants, 1 value of maternal age in each PPV duration subgroup was missing.

2 Factors used to identify matched controls include gestational age, biological sex, mode of delivery, and date of birth (within 2 calendar days).

3 Birth weight in the PPV group is missing 1 subject within the PPV > 1 min subgroup.

4 Cord gas available for 185 infants in the PPV group and 188 infants in the control group.

5 Apgar at 1 and 5 min of life in the PPV group have 201 subjects.

### Neonatal resuscitation team

The hospital’s typical neonatal resuscitation team includes one nurse and two medical providers (e.g., pediatric resident, fellow, nurse practitioner, or physician assistant). All first-year residents (interns) are supervised by a senior resident, fellow or experienced advanced practice provider. The neonatal resuscitation team is called to deliveries based on prenatal factors identified as increased risk for resuscitation^9^. All members of the neonatal resuscitation team are trained in the Neonatal Resuscitation Program^11^ and clinical decisions surrounding resuscitative interventions are based on the AAP/AHA neonatal resuscitation guidelines^9^. In addition to the neonatal team, all Labor and Delivery nurses are trained in the Neonatal Resuscitation Program and may initiate neonatal resuscitation before the team’s arrival. In this study, neonatal team presence captured the team’s presence at any point during resuscitation. During the study period, pulse oximetry was the primary monitor used during neonatal resuscitation when necessary.

### Data collection and definitions

All data were abstracted from the medical record. We collected baseline maternal and neonatal characteristics that may be associated with relevant outcomes after delivery. Maternal obesity was considered if BMI > 30 kg/m^2^ or a diagnosis of obesity was documented in the maternal record. Maternal thyroid dysfunction was defined as active treatment for hypo-or hyperthyroidism. Maternal medication associated with neonatal hypoglycemia after birth was a composite exposure including beta-blockers, systemic antenatal steroids, and medical management for diabetes. Chorioamnionitis was defined based on documentation of the clinical diagnosis of chorioamnionitis by the obstetrical team. Pregnancy complications included oligo- and polyhydramnios, intrauterine growth restriction, and minor congenital anomalies. Minor congenital anomalies include minor heart defects, urinary tract defects, incidental brain findings (calcifications, ventriculomegaly etc.), and dilated colon. The Sepsis Risk Score was calculated at birth based on the Kaiser Permanente calculator^12^.

The primary outcome was neonatal intensive care unit (NICU) admission at any point during the birth hospitalization. In our hospital, infants born ≥35 weeks’ gestation and ≥2 kg are admitted to the newborn nursery unless NICU admission is warranted. Continuous vital sign monitoring and respiratory support are not performed in the newborn nursery; infants who require this level of care are admitted to the NICU. For newborn infants with physiologic stability, interventions provided in the newborn nursery include intravenous antibiotic administration, dextrose gel, and phototherapy.

All patients who require ongoing respiratory support after initial DR resuscitation are admitted to the NICU. For newborn infants who are successfully weaned from respiratory support to room air in the DR, the neonatal medical provider determines the admission destination unit (NICU vs. newborn nursery) based on clinical assessment. No standard protocol for NICU admission based on duration or type of resuscitation exists for these infants.

Secondary outcomes included additional DR interventions, including duration and settings for respiratory support, intubation, chest compressions and epinephrine administration. Duration of PPV was abstracted from the medical documentation. Records where PPV was described subjectively as brief (e.g., couple insufflations, couple breaths, 5–10 breaths) were coded as <1 min. CPAP during resuscitation included provision of CPAP before or after PPV administration until transfer from the DR.

Hospital morbidities were assessed throughout the initial birth hospitalization. They included any respiratory support after the DR, antibiotic therapy, phototherapy, treatment for hypoglycemia (including dextrose gel or intravenous glucose), pneumothorax (based on clinical documentation), culture-proven sepsis, hypoxic-ischemic encephalopathy (defined by clinical exam using the Sarnat score^13^), death before discharge, transfer to a hospital with higher level of care, and length of hospital stay.

In post-hoc analysis, we generated a composite outcome of “any post-delivery complication” after the DR. This included both interventions (respiratory support, phototherapy, treatment for hypoglycemia) and diagnoses (pneumothorax, culture-proven sepsis and hypoxic-ischemic encephalopathy) that require medical attention. Empiric antibiotic administration without culture-proven sepsis was not included in this composite outcome.

### Statistical analysis

Baseline characteristics and neonatal outcomes were summarized using descriptive statistics with representation as mean (standard deviation (SD)) or median (interquartile range (IQR)) for continuous variables and percentages for categorical variables. Normal distribution was assessed using the Shapiro-Wilk test (*P* < 0.05). Missing data from medical records was notified in the tables and statistics were done with the modified group sum.

Baseline characteristics and clinical outcomes were compared between the PPV-exposed infants and controls. We further compared outcomes between infants based on the duration of PPV, defined as ≤1 min versus >1 min PPV. One minute duration of PPV was selected as the cut point based on previous local data demonstrating that approximately half of DR PPV episodes for late preterm and term infants had a duration of PPV < 60 seconds^3^. Rates of any post-delivery complication were compared across control, short-term PPV, and long-term PPV cohorts.

For all comparisons, chi-square and Fisher’s exact tests were used to compare categorical variables. Two-tailed unpaired *t*-test or Mann-Whitney *U* test was used to compare continuous variables. Significance was defined as *P* < 0.05. All analyses were performed using GraphPad Prism version 9.4.1 for Mac, GraphPad Software, San Diego, California, USA.

## Results

Of 3903 infants ≥35 weeks‘ gestation born in 2019 in our center, 233 infants (6%) received PPV in the delivery room, and 202 infants met study eligibility criteria. Most baseline characteristics for PPV-exposed infants were similar to controls who received no respiratory intervention (Table 1). Among infants treated with PPV, 77 (38.1%) received ≤1 min PPV, and 125 (61.9%) received >1 min PPV. (Table 1). Compared with controls, infants who received PPV were more likely to be exposed to chorioamnionitis, meconium-stained amniotic fluid, induced vaginal delivery, instrumentation during labor, urgent C-section, maternal general anesthesia and nuchal cord (Table 1). None of the pregnancy and delivery characteristics differed based on PPV duration.

Table 2 contains resuscitation characteristics for infants who received PPV. Advanced resuscitation measures including intubation, cardiac compression and epinephrine administration were rare and only occurred among infants who received >1 min PPV. While most infants who received PPV left the DR without respiratory support, this was more common for infants who received a brief (≤1 min) duration of PPV.

**Table 2:** Resuscitation Characteristics.

	PPV ≤ 1 min (*n* = 77)	PPV > 1 min (*n* = 125)	*P*-value
CPAP, *n* (%)	54 (70.1%)	104 (83.2%)	0.03
CPAP duration, minutes (median, IQR)	5 (2, 25)	10 (5, 24)	0.06
Maximum PEEP, cmH_2_O (median, IQR)^1^	6 (5, 6)	6 (5, 6)	0.90
PPV Characteristics			
PPV duration, minutes (median, IQR)	0.75 (0.5, 1)	3 (2, 5)	<0.001
Maximum PIP, cmH_2_O (median, IQR)^2^	20 (20, 20)	20 (20, 22)	<0.001
Maximum PEEP, cmH_2_O (median, IQR)^3^	5 (5, 5)	5 (5, 5)	<0.001
Maximum FiO_2_, % (median, IQR)	21 (21, 35)	30 (21, 50)	<0.001
Intubation, *n* (%)	0	9 (7.2%)	N/A
Cardiac compression, *n* (%)	0	1 (0.8%)	N/A
Epinephrine administration, *n* (%)	0	1 (0.8%)	N/A
Final respiratory status leaving the DR			0.002
Intubated, *n* (%)	0 (0%)	9 (7.2%)	
CPAP, *n* (%)	10 (13%)	33 (26.4%)	
No support, *n* (%)	67 (87%)	83 (66.4%)	

CPAP: Continuous positive airway pressure, IQR: Interquartile range, PEEP: Positive end expiratory pressure, PPV: Positive pressure ventilation, PIP: Peak inspiratory pressure, FiO_2_: Fraction of inspired oxygen, N/A: not applicable, DR: Delivery room.

1 Maximum CPAP PEEP is available for 182 infants.

2 Maximum PPV PIP available for 141 infants.

3 Maximum PPV PEEP available for 140 infants.

Following resuscitation, 33.5% of infants in the PPV cohort versus 1.5% in the control cohort were initially admitted to the NICU (Table 3). Transfer rates to the NICU after initial admission to the newborn nursery were low and similar between PPV-exposed infants and controls. Duration of hospital stay was longer in the PPV cohort compared with controls (Table 3). Among infants admitted to the NICU, the duration of NICU admission was longer in the PPV cohort (3 days) compared with controls (2 days, *p* = 0.01). Newborn nursery length of stay was similar between study groups (*p* = 0.3). Within the PPV cohort, the hospital length of stay did not significantly differ based on PPV duration.

**Table 3:** Outcomes and Hospital Morbidities After Delivery Room Resuscitation.

Admission status	PPV (*n* = 202)	Controls (*n* = 202)	*P*-value	PPV ≤ 1 min (*n* = 77)	PPV > 1 min (*n* = 125)	*P*-value
Any NICU admission, *n* (%)	75 (37.1%)	13 (6.4%)	<0.001	20 (26%)	55 (44%)	0.01
Initial NICU admission, *n* (%)^1^	67 (33.5%)	3 (1.5%)	<0.001	15 (19.7%)	52 (41.9%)	<0.001
Any Newborn nursery admission, *n* (%)	135 (66.8%)	199 (98.5%)	<0.001	62 (80.5%)	73 (58.4%)	<0.001
Transfer to NICU, *n* (%)^2^	8 (4%)	10 (5%)	0.63	5 (6.5%)	3 (2.4%)	0.15
Length of stay						
Total hospital stay, days (median, IQR)	3 (2, 4)	2 (2, 3)	<0.001	3 (2,3)	3 (2,4)	0.24
Length of stay after vaginal delivery, days (median, IQR)	2 (2, 2)	2 (2, 2)	<0.001	2 (2, 2)	2 (2, 3)	>0.99
Length of stay after C-section, days (median, IQR)	3 (3, 4)	3 (3, 3)	0.01	3 (2,4)	3 (3,4)	0.09
Hospital outcomes						
Treatment for hypoglycemia, *n* (%)	43/134 (32.1%)	20/87 (23%)	0.14	13/41 (31.7%)	30/93 (32.3%)	0.95
Dextrose gel, *n* (%)	28 (13.9%)	17 (8.4%)	0.08	11 (14.3%)	17 (13.6%)	0.89
IV glucose, *n* (%)	20 (10%)	9 (4.5%)	0.03	4 (5.2%)	16 (12.8%)	0.08
Phototherapy, *n* (%)	16 (7.9%)	11 (5.5%)	0.32	4 (5.2%)	12 (9.6%)	0.26
Duration of Phototherapy, days (median, IQR)	1 (1, 2)	1 (0, 1)	0.02	1.5 (1, 2.8)	1 (1, 2)	0.69
Antibiotics, *n* (%)	42 (20.8%)	3 (1.5%)	<0.001	7 (9.1%)	35 (28%)	0.001
Culture positive sepsis, *n* (%)	0	1 (0.5%)	N/A	0	0	N/A
HIE, *n* (%)	14 (6.9%)	0	N/A	0	14 (11.2%)	N/A
Mild HIE, *n* (%)	1 (0.5%)			0	1 (0.8%)	
Moderate HIE, *n* (%)	9 (4.5%)			0	9 (7.2%)	
Severe HIE n (%)	3 (1.5%)			0	3 (2.4%)	
Any respiratory support, *n* (%)	63 (31.2%)	2 (1%)	<0.001	12 (15.6%)	51 (40.8%)	<0.001
Non-invasive respiratory support, *n* (%)	51 (25.3%)	2 (1%)		11 (14.3%)	40 (32%)	
Invasive respiratory support, *n* (%)	12 (5.9%)	0		1 (1.3%)	11 (8.8%)	
Pneumothorax, *n* (%)	6 (3%)	0	N/A	2 (2.6%)	4 (3.2%)	0.81
Intervention for Pneumothorax, *n* (%)	3 (1.5%)	0	N/A	1 (1.3%)	2 (1.6%)	0.86

DR: Delivery room, PPV: Positive pressure ventilation, NICU: neonatal intensive care unit, IQR: Interquartile range, IV: intravenous, IQR: Interquartile range, HIE: Hypoxic ischemic encephalopathy, N/A: not applicable.

1 In the PPV group, documented indications for initial NICU admission included respiratory distress (54), hypotonia (6), Hypoglycemia (2), suspicion of infection (2) and other (7). In the control group, documented indications for initial NICU admission included respiratory distress (2) and hypoglycemia (1).

2 Eight infants were transferred to the NICU after initial admission to the nursery in the PPV group; indications for transfer included respiratory distress (4), hypoglycemia (1), sepsis evaluation (2) and arrhythmia (1). There were 7 control infants transferred to the NICU for hypoglycemia, 2 for respiratory distress and 1 for arrhythmia.

**Fig. 1:**
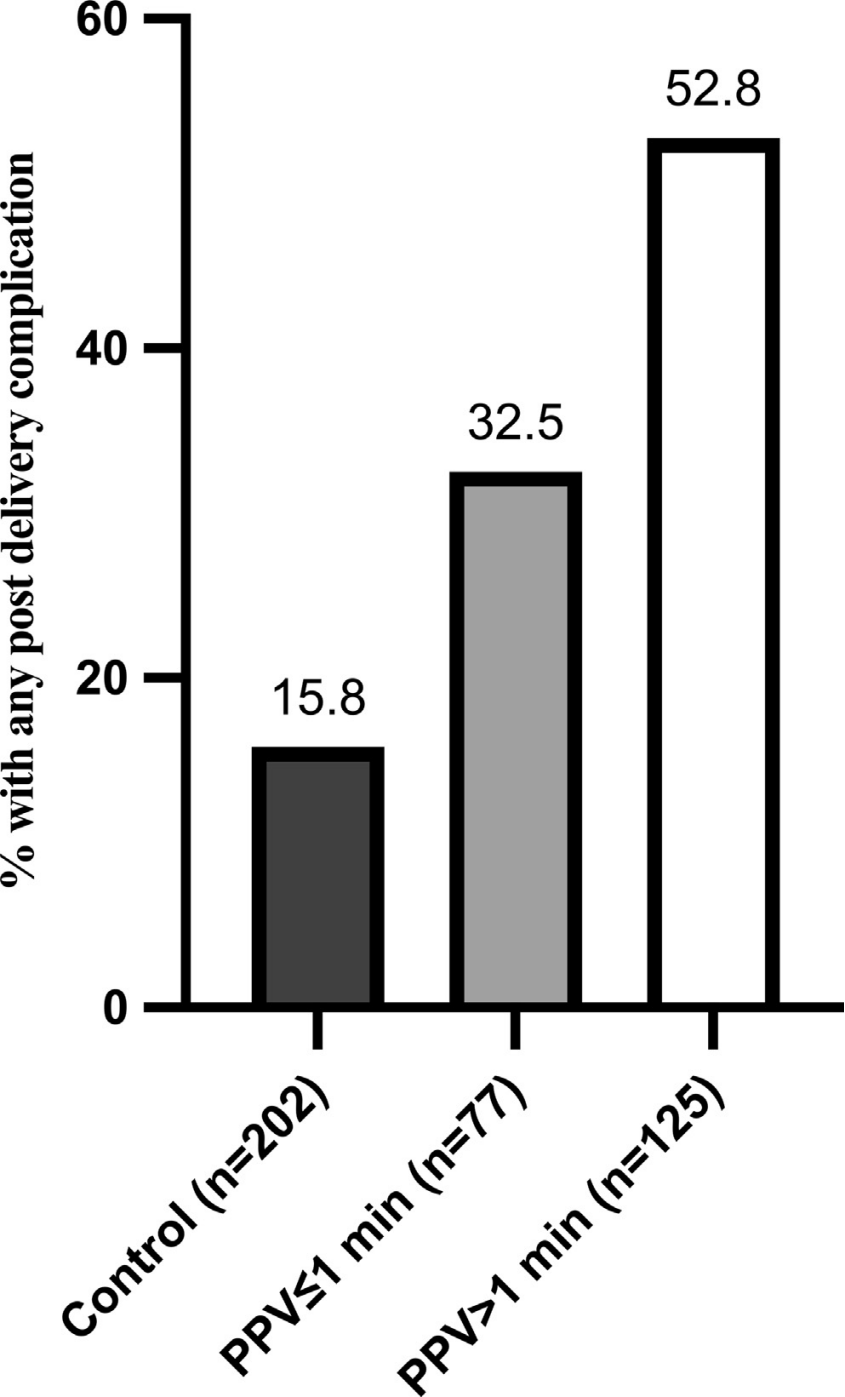
Composite of any post-delivery complication across study groups. The composite outcome of any post-delivery complication includes respiratory support, phototherapy, treatment for hypoglycemia, pneumothorax, culture-proven sepsis and hypoxic-ischemic encephalopathy. Composite outcome rates differed between groups, *p* < 0.0001.

Short-term hospital outcomes and hospital morbidities are shown in Table 3. The composite outcome of any post-delivery complication occurred in 91 (45%) of PPV-exposed infants, compared to 32 (15.8%) of control infants (*p* < 0.001); this composite outcome was more common following >1 min PPV (52.8%) than ≤1 min PPV (32.5%), *p* = 0.002. Rates of this composite outcome differed among all 3 groups examined (Fig. 1). In the PPV cohort, 7 infants were transferred to another hospital for specialized patient care; no control infants were transferred.

## Discussion

We reported short-term outcomes of late preterm and term infants who received DR PPV. Infants who received PPV were more likely to be admitted to the NICU immediately after resuscitation and experienced longer duration of hospital stay and increased frequency of hospital-based morbidities compared with infants who did not receive PPV. However, among infants initially admitted to the newborn nursery, previous exposure to DR PPV was not associated with increased subsequent NICU transfer. Among infants who received PPV, duration of PPV > 1 min was associated with increased antibiotic exposure, hypoxic ischemic encephalopathy and subsequent respiratory support after the DR.

In our cohort, 6% of infants at or above 35 weeks’ gestation received PPV in the DR. These results are consistent with previous literature reporting PPV after birth in 3–10% of newborn infants 3, 4, 5, 10, 14, 15. Within the PPV cohort, 61.9% of infants received >1 min PPV in the DR, comparable to 57% found by Akinloye et al.^5^ and 67% found by Niles et al.^3^

Post-resuscitation monitoring following neonatal resuscitation is recommended.^11^, ^16^ However, there are no uniform recommendations for location, intensity, or type of monitoring for infants who receive DR PPV. Some centers admit all infants who receive DR PPV to the NICU^5^, while others provide oxygen therapy for short durations in the newborn nursery^14^. In our study, 33.5% of infants who received DR PPV were initially admitted to the NICU after resuscitation. This rate is slightly lower than Bjorland et al.,^15^ who reported 46% of infants ≥34 weeks’ gestation who received DR PPV were admitted to the NICU. Differences in the gestational age range for eligible subjects may account for this discrepancy.

Among infants initially admitted to the newborn nursery, NICU transfer rates were low and similar between the PPV cohort and controls. Causes for transfer were primarily respiratory distress in the PPV cohort compared to hypoglycemia in the control cohort. This transfer rate to the NICU is lower than previously published data, which ranges 7.5–39%.^6^, ^14^, ^17^ Potential reasons for the different transfer rates include variability in hospital protocols for post-resuscitation admission and monitoring, differences in the type of support available in each unit, and variability in study design with regards to ascertainment period for this outcome. In our study, the low transfer rate to the NICU suggests DR providers were able to appropriately identify infants who were physiologically stable and unlikely to require further support after transition.

Few studies have compared neonatal outcomes of infants with PPV in the DR with control infants. Two small studies (*n* = 27–33 infants with PPV) demonstrated increased rates of neonatal complications after DR PPV compared with controls^6^, ^17^. In a large single center study by Akinyole et al.^5^ neonatal complications were more common in infants with DR PPV > 1 min compared to shorter PPV; complications were also more common among infants with DR PPV compared to no PPV. However, that study could not assess the association between DR PPV and NICU admission, as hospital policy dictated NICU admission for all infants who received PPV.

We aimed to describe the burden of post-delivery complications among infants who receive PPV, as this may inform policies, practice, and staffing models regarding post-resuscitation care for newborn infants. As anticipated, the controls were generally healthier at birth and experienced fewer morbidities. The control population in our study served to quantify how rates of hospital morbidities following DR-PPV compare with outcomes of newborn infants who do not receive DR-PPV.

While NICU admission was our primary outcome of interest, admission practices at our hospital may not align with other hospitals. The post-hoc “any post-delivery complication” composite outcome was generated to address this limitation. We included diagnoses and interventions in this outcome that required medical attention, regardless of patient location. In our study, infants who received DR PPV, even transiently, more frequently experienced the composite outcome of any post-delivery complication. Importantly, many outcomes such as hyperbilirubinemia requiring phototherapy and hypoglycemia were diagnosed and treated in the newborn nursery. This suggests that post-delivery monitoring while avoiding maternal separation is possible in many cases and may inform post-resuscitation policies that maximize patient safety and limit parental infant separation. Further studies are necessary to determine precise indicators for NICU admission.

### Limitations

We acknowledge limitations inherent to the retrospective and single-center study design and limited study period. Some observed differences between groups with statistical significance may not have clinical relevance. Neonatal providers administered DR PPV based on clinical judgment grounded in the AAP/AHA neonatal resuscitation guidelines. PPV duration was often documented, but some records had an approximation of short PPV administration that was operationalized as less or equal to one minute of PPV in this study, which may have added imprecision. Furthermore, the cutoff between long and short PPV administration was empirically defined at one minute; this is consistent with previous literature but is ultimately an arbitrary threshold. Finally, all patients on respiratory support at the end of resuscitation are admitted to the NICU in our hospital, introducing confounding by indication for the primary outcome. However, this subgroup represents the minority of the PPV cohort, as 150/202 (74%) of PPV-exposed infants were not on any respiratory support at the end of resuscitation.

### Strengths

One of the major strengths of this study is the inclusion of a large cohort of 404 infants born the same year in the same center reducing DR resuscitation protocol variability. Infants were admitted to the NICU based on clinical assessment rather than automatic protocols mandating NICU admission. In addition, this is one of the first studies to compare detailed hospital outcomes for newborn infants who received PPV with similar controls who received no DR resuscitation. Finally, this is one of the few studies to examine the association of PPV duration in the DR with short-term outcomes after the DR.

## Conclusion

Late preterm and term infants are at increased risk for NICU admission and postnatal complications following DR PPV. These results suggest that post-resuscitative monitoring in the NICU or newborn nursery is essential after DR PPV, regardless of PPV duration. Prospective studies are needed to identify the optimal location and type of monitoring for newborn infants following DR resuscitation including PPV.

## Funding

This work was supported by the Belgian French Community (Fund for Scientific Research) through a FRIA scholarship benefitting Maureen Peers de Nieuwburgh and the AHRQ career development award (K08HS029029) supporting Heidi Herrick.

## CRediT authorship contribution statement

**Maureen Peers de Nieuwburgh:** Writing – review & editing, Writing – original draft, Software, Methodology, Investigation, Formal analysis, Data curation, Conceptualization. **Charlotte Cecarelli:** Writing – review & editing, Data curation, Conceptualization. **Danielle Weinberg:** Writing – review & editing, Supervision, Software, Resources, Project administration, Methodology, Funding acquisition, Data curation, Conceptualization. **Kesi C. Yang:** Writing – review & editing, Validation, Supervision, Resources. **Heidi M. Herrick:** Writing – review & editing, Validation, Supervision, Resources. **Elizabeth E. Foglia:** Writing – review & editing, Validation, Supervision, Methodology, Investigation, Formal analysis, Conceptualization.

## Declaration of competing interest

The authors declare the following financial interests/personal relationships which may be considered as potential competing interests: ‘This work was supported by the Belgian French Community (Fund for Scientific Research) through a FRIA scholarship benefitting Maureen Peers de Nieuwburgh and the AHRQ career development award (K08HS029029) supporting Heidi Herrick. The funding sources had no involvement in study design, data collection, analysis and interpretation, writing of the report and in the decision to submit the article for publication. Additionally, we confirm there is no overlap with previous publications and that these findings are novel and have not been published or submitted elsewhere. There is no other conflict of interest to disclose’.
